# Novel Optical Coherence Tomography Parameters as Prognostic Factors for Stage 3 Epiretinal Membranes

**DOI:** 10.1155/2020/9861086

**Published:** 2020-12-22

**Authors:** Young Gun Park, Seo Yeon Hong, Young-Jung Roh

**Affiliations:** ^1^Department of Ophthalmology and Visual Science, Seoul St. Mary's Hospital, College of Medicine, The Catholic University of Korea, Seoul, Republic of Korea; ^2^Department of Ophthalmology and Visual Science, Yeoido St. Mary's Hospital, College of Medicine, The Catholic University of Korea, Seoul, Republic of Korea

## Abstract

**Purpose:**

We aimed to describe the visual prognosis of eyes with ectopic inner foveal layers (EIFLs) after epiretinal membrane (ERM) surgery.

**Methods:**

This retrospective study enrolled patients diagnosed with stage 3 ERM based on the EIFL staging scheme who underwent ERM surgery with a minimum follow-up period of 12 months. Central foveal thickness (CFT), EIFL thickness, and the length of the ellipsoid zone defect were evaluated at baseline and at 1 month, 6 months, and 12 months after surgery based on pre- and postoperative swept-source optical coherence tomography (OCT) images. The association of EIFL thickness and other OCT parameters with pre- and postoperative best-corrected visual acuity (BCVA) was analyzed.

**Results:**

Sixty-nine eyes with stage 3 ERMs were analyzed. Preoperative BCVA was correlated with preoperative CFT (*r* = 0.517, *p* < 0.001) and preoperative EIFL thickness (*r* = 0.652, *p* < 0.001). At 12 months, postoperative BCVA was correlated negatively with preoperative CFT (*r* = 0.470, *p*=0.016) and preoperative EIFL thickness (*r* = 0.582, *p*=0.004). The improvement in BCVA was not associated with postoperative reduction in CFT (*p*=0.06), although it was significantly associated with postoperative reduction in EIFL thickness (*r* = 0.635, *p*=0.007).

**Conclusions:**

EIFL thickness should be considered a negative prognostic factor for postoperative anatomical and functional recovery in patients with stage 3 ERMs.

## 1. Introduction

The epiretinal membrane (ERM) is a common macular disorder characterized by fibrocellular proliferation on the inner retinal surface, which causes morphologic distortion and affects central vision [1]. ERM cases that involve decreased or distorted central vision require treatment with surgical procedures such as pars plana vitrectomy with ERM peeling. Microincision vitrectomy has been widely used recently and has shown higher rates of anatomical success [2, 3]. However, these anatomical outcomes do not correspond with better visual prognosis. Visual prognostic factors for ERM surgery, using spectral-domain optical coherence tomography (OCT), have consequently been published [4–7].

Previously published visual prognostic spectral-domain OCT findings regarding ERM surgery may be divided into inner and outer segment factors. The inner segment factor associated with poor visual prognosis after ERM surgery is foveal inner retinal layer thickness [8, 9]; outer segment factors associated with poor visual prognosis include an outer nuclear complex, cone outer segment tip defect length, and ellipsoid zone (EZ) defect length [10, 11].

**Figure 1:**
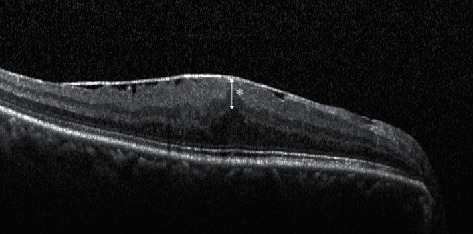
An ectopic inner foveal layer (EILF). The EIFL (asterisk) on the optical coherence tomography image indicates the presence of continuous hyporeflective and hyperreflective bands extending from the inner nuclear layer and inner plexiform layer across the foveal region.

Govetto et al. [12] recently suggested a new OCT-based grading system to classify ERMs based on the presence of a continuous ectopic inner foveal layer (EIFL) as a new finding in advanced stages (i.e., stages 3 and 4). They also suggested the presence of the “central bouquet” on OCT images that refers to a foveal bulge at the level of the outer retina. Stage 3 was defined as the presence of an ERM with a continuous EIFL, whereas stage 4 was defined as significant retinal thickening with anatomical disruptions in the macula (Figure 1).

Previous reports have described relative associations between inner retinal thickness, EIFL thickness, and postoperative visual acuity in idiopathic ERMs [13, 14]. However, EIFL thickness measurements in patients with stage 4 ERMs are unreliable because of the remarkable preoperative disruption of the retinal layers. Therefore, we excluded patients with stage 4 ERMs. The aim of the current study was to investigate the relationship between OCT parameters and visual prognosis in stage 3 ERM patients who underwent 25-gauge vitrectomy.

## 2. Materials and Methods

### 2.1. Study Design

In this study, we retrospectively reviewed data of consecutive patients who presented to the Department of Ophthalmology of Seoul St. Mary's Hospital, Korea, between January 2018 and January 2019 with a confirmed diagnosis of primary idiopathic stage 3 ERM and were treated with 25-gauge vitrectomy for ERM with indocyanine green- (ICG-) assisted internal limiting membrane peeling by a single surgeon (YGP).

All procedures were conducted according to the tenets of the Declaration of Helsinki and its later amendments. The study was approved by the ethics committee of Seoul St. Mary's Hospital and the Catholic University of Korea. The need to obtain informed patient consent was waived because of the retrospective study design.

Patients who underwent ERM removal surgery for unilateral idiopathic stage 3 ERM and have been followed at least 12 months after surgery were included. Those with stage 4 ERM, secondary or bilateral ERM, and any other ocular disease that could affect visual function (e.g., glaucoma, age-related macular degeneration, and refractive error >5 diopters), severe media opacity (e.g., lens opacity owing to cataract or thick asteroid hyalosis), or those lost to the follow-up after ERM surgery were excluded.

All patients and controls initially underwent measurement of their best-corrected visual acuity (BCVA) using the standard Snellen chart. The results were converted to the logarithm of the minimal angle of resolution (logMAR) values for statistical analysis. The patients then underwent standardized fundus examination, which included measurements using swept-source OCT (DRI OCT Triton; Topcon, Tokyo, Japan). SS-OCT was performed before and at 1, 6, and 12 months after surgery.

### 2.2. Swept-Source OCT Imaging and OCT Parameters

Swept-source OCT utilizes a wavelength of 1,050 nm and reaches a scanning speed of 100,000 A-scans per second, with 8 *μ*m and 20 *μ*m axial and transverse resolution in tissue [15]. The devices produce OCT B-scan images derived from 512 × 256 axial scans over a scan area of 12 × 9 mm^2^. This high-quality fundus imaging technique relies on active eye tracking. Only images with a quality score of more than 60 were included. We used image viewer software (IMAGEnet 6, version 1.24; Topcon), and the thickness was determined by consensus between two observers (YGP and YJR) who were blinded to all clinical information.

OCT parameters included central foveal thickness (CFT), outer nuclear layer thickness (ONL), EIFL thickness, and length of the EZ defect. When the foveal depression was absent, the foveal center was identified by the point of the greatest outer nuclear layer thickness and the bulge-like structure of the IS/OS junction at the fovea. The ONL thickness measured from the inner border of the retinal pigment epithelium to the border of the ONL, and the EIFL thickness, defined as the distance between the inner border of the ONL and the ILM at the foveal center [16]. The EZ defect was considered to be the extent with the loss of the hyperreflective signal that characterizes the layer at the horizontal one passing through the fovea [17]. Previous reports have demonstrated a relationship between vision loss associated with ERM and disruption of the EZ and outer photoreceptor segments [9, 18]. Disruption of the EZ has been widely recognized to be related with visual prognosis in various macular diseases, such as macular holes and edema [19–21]. We therefore measured the length of EZ disruption.

### 2.3. Surgical Treatment

All surgeries were performed by a single surgeon (YGP). A three-port 25-gauge transconjunctival sutureless vitrectomy was performed to remove the ERM. After vitrectomy, the ERM was removed using end-gripping forceps (Alcon, Fort Worth, TX, USA). After removing the ERM, internal limiting membrane peeling was performed with 0.25% indocyanine green dye. ERM and internal limiting membrane peeling were started at the outer region around the fovea, particularly in the parafoveal area.

### 2.4. Statistical Analysis

The normal distribution of data was assessed using the Shapiro–Wilk test. For normally distributed data, Pearson's correlation and regression tests were performed. The analysis of variance was performed for each parameter. For nonparametric data, Spearman's rank correlation test was used. All analyses were conducted using SPSS (IBM SPSS Statistics, version 24.0; IBM Corporation, New York, NY, USA). A *p* value < 0.05 was statistically significant.

## 3. Results

We reviewed clinical records of 69 patients diagnosed with stage 3 ERMs; the patients comprised 25 (36.2%) men and 44 (63.8%) women. The mean age of the patients was 67.78 ± 6.69 years; 52 of 69 eyes (75.4%) exhibited mild cataract (2.06 ± 0.64 using the lens opacity classification (LOCS III) scale) [22] and underwent combined phacoemulsification. The mean preoperative BCVA was 0.47 ± 0.16 logMAR, and the mean CFT was 480.08 ± 60.47 *µ*m. The mean EIFL thickness was 183.41 ± 89.50 *µ*m, and the mean ONL was 163.04 ± 46.3 *µ*m; the mean EZ defect length was 480.3 ± 162.1 *µ*m. Baseline characteristics of the patients at presentation are summarized in Table 1.

### 3.1. Preoperative Visual Acuity and OCT Parameters

The preoperative BCVA correlated with preoperative CFT (*r* = 0.517, *p* < 0.001) and preoperative EIFL thickness (*r* = 0.652, *p* < 0.001). The preoperative CFT was relatively strongly correlated with preoperative EIFL thickness (*r* = 0.54, *p*=0.001). Thus, these variables shared an effect on BCVA. On multiple regression analysis, only EIFL thickness (*p* < 0.001) was significantly associated with worse preoperative BCVA.

### 3.2. Postoperative Visual Acuity and OCT Parameters

The mean preoperative and postoperative BCVA at 1, 6, and 12 months are listed in Table 2. The CFT and EIFL thickness also significantly decreased at 1, 6, and 12 months postoperatively, as shown in Table 2. However, the ONL thickness and length of the EZ defect did not show a significant difference (all, *p* > 0.05).

**Figure 2:**
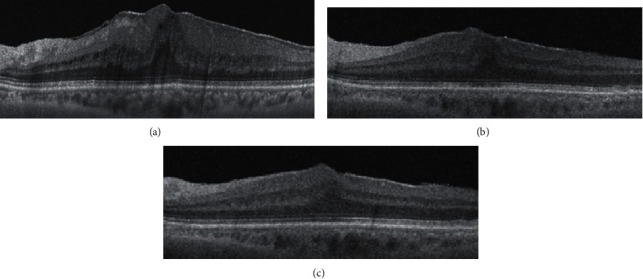
Optical coherence tomography images from a patient diagnosed with an epiretinal membrane (ERM). (a) Stage 3 ERM was diagnosed based on swept-source optical coherence tomography findings: the central fovea contains continuous ectopic inner foveal layers (EIFLs). (b) At 1 month after surgery, a thick EIFL persists over the outer nuclear layer. (c) At 12 months after surgery, the EIFL persists, although significant thinning has occurred. Visual acuity changed from 0.39 logMAR to 0.1 logMAR at 12 months after ERM surgery.

**Figure 3:**
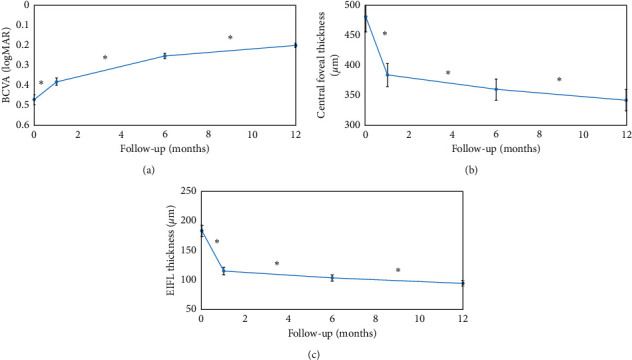
Functional and anatomical changes occurring from baseline to the 12 months postoperatively. (a) The best-corrected visual acuity (BCVA) significantly improved in the postoperative follow-up period (*p* < 0.001). (b) In the follow-up period, the central foveal thickness (CFT) decreased significantly with a noticeable effect at 1 month after surgery (*p*=0.001). (c) Similar to the CFT, the thickness of the ectopic inner foveal layers (EIFLs) decreased significantly with a prominent effect in the first month after surgery (*p*=0.003).

**Figure 4:**
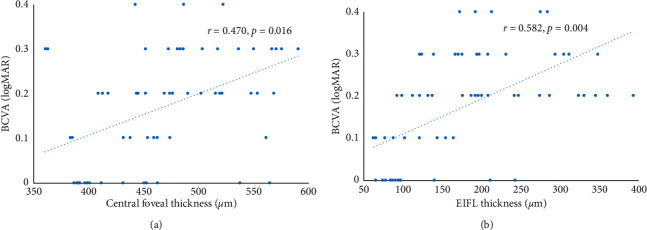
a, b Correlation analysis between postoperative best-corrected visual acuity (BCVA) and the central foveal thickness (CFT) and ectopic inner foveal layer (EIFL) thickness at 12 months after surgery. Optical coherence tomography parameters are significantly associated with postoperative BCVA (CFT: r = 0.470 and *p*=0.016; EIFL: r = 0.582 and *p*=0.004). logMAR, logarithm of the minimum angle of resolution.

Postoperative changes in EIFL thickness and CFT were greatest in the first month after surgery, and the postoperative BCVA continued to improve slightly until 12 months postoperatively (Figure 2). The postoperative BCVA improved gradually until the end of the follow-up period (Figures 3 and 4). At 12 months, the postoperative BCVA correlated negatively with the preoperative CFT (*r* = 0.470, *p*=0.016) and preoperative EIFL thickness (*r* = 0.582, *p*=0.004). However, the ONL thickness and length of the EZ defect showed no significant difference (all, *p* > 0.05) (Figure 4).

To identify OCT parameters whose improvement after ERM surgery was associated with visual improvement, a correlation analysis was conducted between the amount of postoperative BCVA improvement and changes in OCT parameters. BCVA improvement was not associated with postoperative CFT reduction (*p*=0.06), although it was significantly associated with a postoperative decrease in EIFL thickness (*r* = 0.635, *p*=0.007).

At 12 months after surgery, the EIFL persisted postoperatively in most patients and was present in 54 (78.3%) of 69 eyes. Cotton ball signs existed in 18 (26.1%) of 69 eyes at baseline; however, all of these signs disappeared during the postoperative follow-up period. No serious intra- or postoperative complications were recorded during the follow-up period.

## 4. Discussion

ERM is one of the most common macular diseases, and its prevalence tends to increase with age [23, 24]. Patients with ERM may experience problems such as metamorphopsia and decreased visual acuity. To resolve these symptoms, surgical removal of ERM is recommended as standard treatment [25]. However, the desired visual outcomes are not always achieved, even with apparently successful ERM removal. Clinicians need to measure the severity of ERM and predict parameters for visual prognosis.

Recent advancements in OCT have led to a greater interest in assessing retinal microstructures using this technology. Therefore, the identification of reliable prognostic biomarkers with OCT is important for improving prediction of postoperative outcomes in patients with idiopathic ERMs. Many published spectral-domain OCT studies have demonstrated a relationship between retinal microstructural alterations such as the disruption of EZ or outer photoreceptor segments and vision loss in ERMs [13, 26–28].

The role of the inner retina in visual acuity loss has been studied more closely. Govetto et al. [12] suggested a new OCT-based grading system to classify ERMs, with advanced ERMs showing the presence of a preoperative continuous EIFL. As the ERM stage increases, the progression of this anatomical finding correlates with decreased visual acuity [29]. This factor may also be associated with visual acuity in patients with idiopathic ERM formation. Our study focused on stage 3 ERM to ascertain the influence of EIFL. Patients with stage 4 ERM had an extensive EIFL that covered the entire foveal area. Their retinal layers were noted to be significantly distorted and disorganized and were not clearly identified with OCT. Therefore, we excluded patients with stage 4 ERM and only included cases with stage 3 severity.

Preoperative BCVA correlated with preoperative CFT and preoperative EIFL thickness. However, the preoperative CFT was relatively strongly correlated with preoperative EIFL thickness. These variables shared an effect on BCVA; it may be explained by the fact that the EIFL is a key factor underlying increased CFT. The primary finding of this study was that preoperative CFT thickness and EIFL thickness were significantly associated with poor postoperative visual prognosis in patients with stage 3 ERM. In addition, only preoperative EIFL thickness was significantly associated with BCVA improvement. Thus, inner retinal OCT findings associated with EIFL thickness were more significantly associated with visual prognosis after ERM surgery.

Alkabes et al. [30] demonstrated that EIFL thickness and CFT correlated significantly with metamorphopsia, demonstrated by M-CHARTS in the advanced stages of ERM (stages 3 and 4) based on the OCT-based grading scheme [12] which included a new OCT parameter such as EIFL (both *p* < 0.0001). They only included 37 eyes with advanced ERMs; however, the results indicated that EIFL thickness could be a good indicator for metamorphopsia. Gonzalez-Saldivar et al. [16] used the EIFL staging scheme as a visual prognostic factor and assessed final BCVA based on the stages. They found that earlier stages were associated with better visual outcomes preoperatively and postoperatively in patients undergoing ERM surgery (stage 2 > stage 3 > stage 4, *p* < 0.001). They also noted that surgery in patients with stage 2 ERM results in significantly better visual outcomes. In our study, we also noted that the thickness of the preoperative EIFL was negatively associated with postoperative BCVA.

ERM is an inner retinal disease, and OCT findings showing improvement after surgery are mostly observed in the inner retina. Several recent studies have evaluated inner rather than external retinal biomarkers as prognostic factors for ERM surgery [8, 31–33]. In our study, as an outer biomarker, EZ disruption was not significantly correlated with poor visual prognostic factors. Conversely, the EIFL thickness of the inner retinal OCT parameters was more significantly associated with the visual prognosis of ERM surgery in patients with advanced ERM stages. The EIFL thickness, which is based on OCT images, is a more practical and reproducible tool for obtaining visual prognosis in patients with ERM. Therefore, it is essential that EIFL formation is taken into consideration during decision-making for ERM surgery.

This study had some limitations. First, data collection was performed retrospectively by reviewing medical records. Second, we used a relatively small sample and included patients with and without a history of cataract surgery. Third, VA values may be affected by different degrees of lens opacity. Fourth, OCT images were analyzed by a skilled retinal specialist; however, the use of manual measurements instead of automatically provided absolute values, which could have introduced bias. Finally, we used ICG dye for staining during internal limiting membrane peeling. ICG dye is associated with retinal toxicity; therefore, we attempted to reduce the exposure time to a relatively short duration.

## 5. Conclusions

We observed that the postoperative visual outcome of eyes with stage 3 ERM significantly correlated with preoperative EIFL thickness and CFT at baseline. Moreover, the length of the EZ defect at baseline did not significantly correlate with postoperative visual acuity. These findings may help retinal surgeons determine the surgical indications and optimal timing for surgical treatments. Further clinical studies are required to validate the findings of this study.

## Figures and Tables

**Table 1:** Baseline demographics.

Characteristic	
Number of patients (n)	69
Sex (male : female)	25 : 44
Age (y)	67.78 ± 6.69
Combined cataract surgery	52 (75.4%)
BCVA (logMAR)	0.47 ± 0.16
CFT (*µ*m)	480.08 ± 60.47
ONL thickness (*µ*m)	163.04 ± 46.3
EIFL thickness (*µ*m)	183.41 ± 89.50
Length of the EZ defect (*µ*m)	480.3 ± 162.1
Cotton ball sign	18 (26.1%)

BCVA, best-corrected visual acuity; CFT, central foveal thickness; ONL, outer nuclear layer thickness; EIFL, ectopic inner foveal layer; EZ, ellipsoid zone.

**Table 2:** Comparison of preoperative and postoperative BCVA, CFT, and the thickness of EIFL in patients with idiopathic epiretinal membranes.

Time point	BCVA (logMAR)		CFT (*µ*m)		The thickness of EIFL (*µ*m)	
	Mean ± SD	*p* value^a^	Mean ± SD	*p* value^a^	Mean ± SD	*p*value^a^
Preoperative	0.47 ± 0.16		480.08 ± 60.47		183.41 ± 89.5	
Post-1M	0.38 ± 0.18	0.008^*∗*^	384.03 ± 41.55	<0.001^*∗*^	104.18 ± 46.13	0.012^*∗*^
Post-6M	0.25 ± 0.14	0.001^*∗*^	360.03 ± 44.84	<0.001^*∗*^	93.25 ± 27.1	0.028^*∗*^
Post-12M	0.20 ± 0.13	<0.001^*∗*^	342.63 ± 42.46	<0.001^*∗*^	80.5 ± 37.3	0.003^*∗*^

^a^ *P* value vs. preoperative; BCVA, best-corrected visual acuity; CFT, central foveal thickness; EIFL, ectopic inner foveal layer.

## Data Availability

The data used to support the findings of this study are available from the corresponding author upon request.
